# *Streptococcus pneumoniae* disrupts the structure of the golgi apparatus and subsequent epithelial cytokine response in an H_2_O_2_-dependent manner

**DOI:** 10.1186/s12964-023-01233-x

**Published:** 2023-08-17

**Authors:** Björn Klabunde, André Wesener, Wilhelm Bertrams, Stephan Ringshandl, Luke D. Halder, Evelyn Vollmeister, Bernd Schmeck, Birke J. Benedikter

**Affiliations:** 1 Institute for Lung Research, Universities of Giessen and Marburg Lung Center (UGMLC), Philipps-University Marburg, Marburg, Germany; 2 Center for Synthetic Microbiology (SYNMIKRO), Philipps-University Marburg, Marburg, Germany; 3 Institute for Lung Health (ILH), Giessen, Germany; 4 Department of Medicine, Pulmonary and Critical Care Medicine, University Medical Center Marburg, Philipps-University Marburg, Marburg, Germany; 5 Member of the German Center for Lung Research (DZL), German Center for Infectious Disease Research (DZIF), Marburg, Germany; 6 Core Facility Flow Cytometry - Bacterial Vesicles, Philipps-University Marburg, Marburg, Germany; 7 School for Mental Health and Neuroscience, University Eye Clinic Maastricht, Maastricht University Medical Center (MUMC+), Maastricht University, P. Debyelaan 25, 6229 HX Maastricht, The Netherlands

## Abstract

**Background:**

Lung infections caused by *Streptococcus pneumonia* are a global leading cause of death. The reactive oxygen species H_2_O_2_ is one of the virulence factors of *Streptococcus pneumoniae*. The Golgi apparatus is essential for the inflammatory response of a eukaryotic cell. Golgi fragmentation was previously shown to be induced by bacterial pathogens and in response to H_2_O_2_ treatment. This led us to investigate whether the Golgi apparatus is actively involved and targeted in host–pathogen interactions during pneumococcal infections.

**Methods:**

Following in vitro infection of BEAS-2B bronchial epithelial cells with *Streptococcus pneumoniae for* 16 h, the structure of the Golgi apparatus was assessed by fluorescence staining of the Golgi-associated protein, Golgin-97. To investigate the effect of H_2_O_2_ production on Golgi structure, BEAS-2B cells were treated with H_2_O_2_ or the H_2_O_2_ degrading enzyme Catalase, prior to Golgi staining. Artificial disruption of the Golgi apparatus was induced by treatment of cells with the GBF1 inhibitor, Golgicide A. A proinflammatory cellular response was induced by treatment of cells with the bacterial cell wall component and TLR4 ligand lipoteichoic acid.

**Results:**

In vitro infection of bronchial epithelial cells with wild type *Streptococcus pneumoniae* led to a disruption of normal Golgi structure. Golgi fragmentation was not observed after deletion of the pneumococcal H_2_O_2_-producing gene, *spxB*, or neutralization of H_2_O_2_ by catalase treatment, but could be induced by H_2_O_2_ treatment. *Streptococcus pneumoniae* infection significantly reduced host cell protein glycosylation and artificial disruption of Golgi structure significantly reduced bacterial adherence, but increased bacterial counts in the supernatant. To understand if this effect depended on cell-contact or soluble factors, pneumococci were treated with cell-supernatant of cells treated with Golgicide A and/or lipoteichoic acid. This approach revealed that lipoteichoic acid conditioned medium inhibits bacterial replication in presence of host cells. In contrast, artificial Golgi fragmentation by Golgicide A treatment prior to lipoteichoic acid treatment rescued bacterial replication. This effect was associated with an increase of IL-6 and IL-8 in the supernatant of lipoteichoic acid treated cells. The increased cytokine release was abolished if cells were treated with Golgicide A prior to lipoteichoic acid treatment.

**Conclusion:**

*Streptococcus pneumoniae* disrupts the Golgi apparatus in an H_2_O_2_-dependent manner, thereby inhibiting paracrine anti-infective mechanisms.

Video Abstract

**Supplementary Information:**

The online version contains supplementary material available at 10.1186/s12964-023-01233-x.

## Background

*Streptococcus pneumoniae* (*Spn*) is a major agent of community acquired pneumonia, as well as other invasive infectious diseases like otitis media, meningitis and sepsis, thereby causing an estimated 829.000 annual deaths, globally [1]. Typically, pneumococcal disease starts with asymptomatic bacterial colonization of the upper respiratory tract. If the immune system is weakened, the bacteria may advance to the lower respiratory tract and start to replicate in the lung alveoli, causing pneumonia.

*Spn* employs an array of virulence factors in order to successfully adhere to host cells, evade the immune system and progress through the epithelial barrier. Amongst those are an array of often glycosylated surface proteins which mediate adherence to the host epithelium [2–4]. In addition to these surface structures, *Spn* virulence factors comprise soluble factors such as the pore-forming cytolysin pneumolysin and the oxidant H_2_O_2_.

H_2_O_2_ in *Spn* is produced predominantly by the pneumococcal pyruvate oxidase (*spxB*) as a by-product of acetyl-pyruvate production [5]. It then accumulates due to the absence of the H_2_O_2_-degrading enzyme catalase [6]. In host–pathogen interaction during pneumococcal infection, H_2_O_2_ was shown to induce DNA damage and apoptosis in alveolar epithelial cells [7]. It was, furthermore, shown to suppress the NLRP3 and NLRC4 dependent inflammasome and mediate pneumococcal protection against the innate immune response [8].

The lung epithelium constitutes the first line of defense against pneumococcal infection in the lower airways. Upon detection of pathogens, it orchestrates a strong proinflammatory response to recruit immune cells and clear the site of infection. The Golgi apparatus (Golgi) is a host cell organelle with multi-faceted functions. Intracellular trafficking and sorting mediated through the Golgi is vital for the proper execution of the proinflammatory response. Newly synthesized proteins enter the Golgi through the nucleus-facing *cis*-Golgi network. Golgi-membrane associated tethering proteins transport these new proteins towards the *trans*-Golgi cisterna and the *trans*-Golgi network [9]. Tethering proteins within the *trans*-Golgi network then sort the proteins by destination [10]. A second main function of the Golgi is the posttranslational glycosylation of proteins. Glycosyltransferases and are distributed through the Golgi in a defined sequence reaching from the *cis*- to the *trans*-Golgi-network, catalyzing the attachment of glycosylation motifs onto the protein [11]. It was recently shown that the Golgi apparatus exhibits structural damage upon H_2_O_2_ treatment [12].

In this study, we analysed whether *Spn* infection of BEAS-2B lung epithelial cells interferes with Golgi structure and function and found an H_2_O_2_-dependent fragmentation of the Golgi upon *Spn* infection. Artificial disruption of the Golgi resulted in attenuated cytokine secretion and increased bacterial replication, thereby promoting the infection process.

## Material and methods

### Cultivation and stimulation of BEAS-2B cells

The human bronchial epithelial cell line BEAS-2B was obtained from ATCC (CRL-9606) and cultivated in BEGM medium (Lonza, CC-3170) according to ATCC guidelines without fibronectin coating. Approximately 2*10^5^ cells per well were seeded into 24 well-plates (surface area 1.1 cm^2^) and cultivated for 24 h prior to infection/stimulation. For stimulation experiments, medium was exchanged and cells were treated with H_2_O_2_ (Sigma-Aldrich, H1009), 40 or 80 µM for 24 h or with Golgicide A (Tocris, 3584), 4 µM for 4 or 24 h. To generate Golgicide A/lipoteichoic acid (LTA) conditioned supernatants, cells were first treated with Golgicide A, 4 µM for 4 h. Afterwards, medium was exchanged and cells were treated with LTA, 1 µg/ml for additional 2 h and medium collected.

### Bacterial cultivation and infection of BEAS-2B cells

*Spn* strains D39, TIGR4, D39Δ*spxB and* D39Δ*ply* were kindly provided by Sven Hammerschmidt (University of Greifswald). *Spn* was incubated over night at 37 °C / 5% CO_2_ on Columbia Blood agar plates (Becton Dickinson, 254005). Single colonies were inoculated into THY media (30 g/l Todd-Hewitt-Bouillon, Roth, X936.1 and 5 g/l yeast extract, Roth, 2363.2) and incubated at 37 °C / 5% CO_2_ until early logarithmic phase (approx. OD_600_ = 0.33) was reached. For BEAS-2B infection, bacteria were centrifuged (3000xg, 10 min) and inoculated into fresh bronchial epithelial cell growth medium (BEGM) according to desired multiplicity of infection (MOI). Control cells were left untreated. For adherence and internalization assays, BEAS-2B were infected at MOI 20 for 2 h. Infected cells were washed with PBS to remove non-adherent bacteria. Cells were then detached using trypsin and lysed in distilled H_2_O. Dilutions of the cell lysate were plated on blood agar plates to determine CFU.

### Cell viability measurement

MTT assays were performed on 96-well plates to assess cell viability. After stimulation or infection, medium was replaced with fresh medium, supplemented with 0.5 mg/ml MTT reagent (Merck, CT01-5). Cells were incubated for approx. 3 h, until visible dark crystals had formed. Medium was removed and crystals dissolved in 100 µl 1:1 DMSO:EtOH. Absorbance was measured in a plate reader at λ = 570 nm. Viability was assessed as absorbance of treated samples relative to control cells.

### Immuno-fluorescence staining

BEAS-2B cells were seeded and cultivated on 12 mm glass slides. After stimulation or infection, cells were washed with PBS and fixed with 4% paraformaldehyde for 20 min at room temperature. After blocking with 4% milk powder in PBS, cells were incubated with the primary antibody (mouse α Golgin-97, ThermoFisher, 14–9767-82), diluted 1:50 in TBS + 0.35% TritonX-100 for 2 h. Cells were washed with PBS and incubated with the secondary antibody (Goat α mouse, Alexa Fluor 488, ThermoFisher, A-11001; dilution 1:500) and DAPI (1:1000, Sigma, D9542), in TBS + TritonX-100 for 30 min. Cells were washed three times with PBS and once with distilled water and embedded in Mowiol® 4–88 prior to microscopy. Fluorescence images were taken using an AXIO Verta1 microscope (Zeiss) with a TCS SP5 II camera with HCX PL (Leica) at 1000-fold magnification.

### Quantification of the Golgi surface area

For quantification of the Golgi area, we adjusted a method published previously [13]. Fluorescence images were converted into binary images using Fiji ImageJ, V2.9.0: First, the signal intensity over the whole image was determined. A threshold for conversion into a binary image was set so that only signals with an intensity of at least 15% of the maximum signal intensity were included. The whole signal area was then measured as surface area and normalized against the cell count as determined by counting DAPI-stained nuclei.

### Cytokine measurement

Supernatant containing secreted proteins was reduced to 100 µl volume by vacuum evaporation at 30 °C for 7 h. A panel of 13 different cytokines was detected in the concentrated supernatant using the LEGENDplex™ human inflammation panel 1 multiplex FACS kit (BioLegend, Cat: 740809, Lot No. B347171); of these, IL-6, IL-8 and IL-18 were reproducibly detected above quantification threshold. Experiments were performed according to manufacturer's protocol and measured on a BD FACSymphony A1 flow cytometer with BD FACS diva 9.0 software. Results were analyzed using LEGENDplex™ Data Analysis Software Suite.

### Statistics

Data was plotted and analyzed using Prism software V9.5.0 (GraphPad, La Jolla, USA). Data are depicted as mean values + SEM for at least three biologically independent replicates. *P*-values ≤ 0.05 were considered statistically significant and marked with *. Unless indicated otherwise, tests were performed vs. corresponding control.

## Results

### Spn infection induces breakdown of Golgi structure

**Fig. 1:**
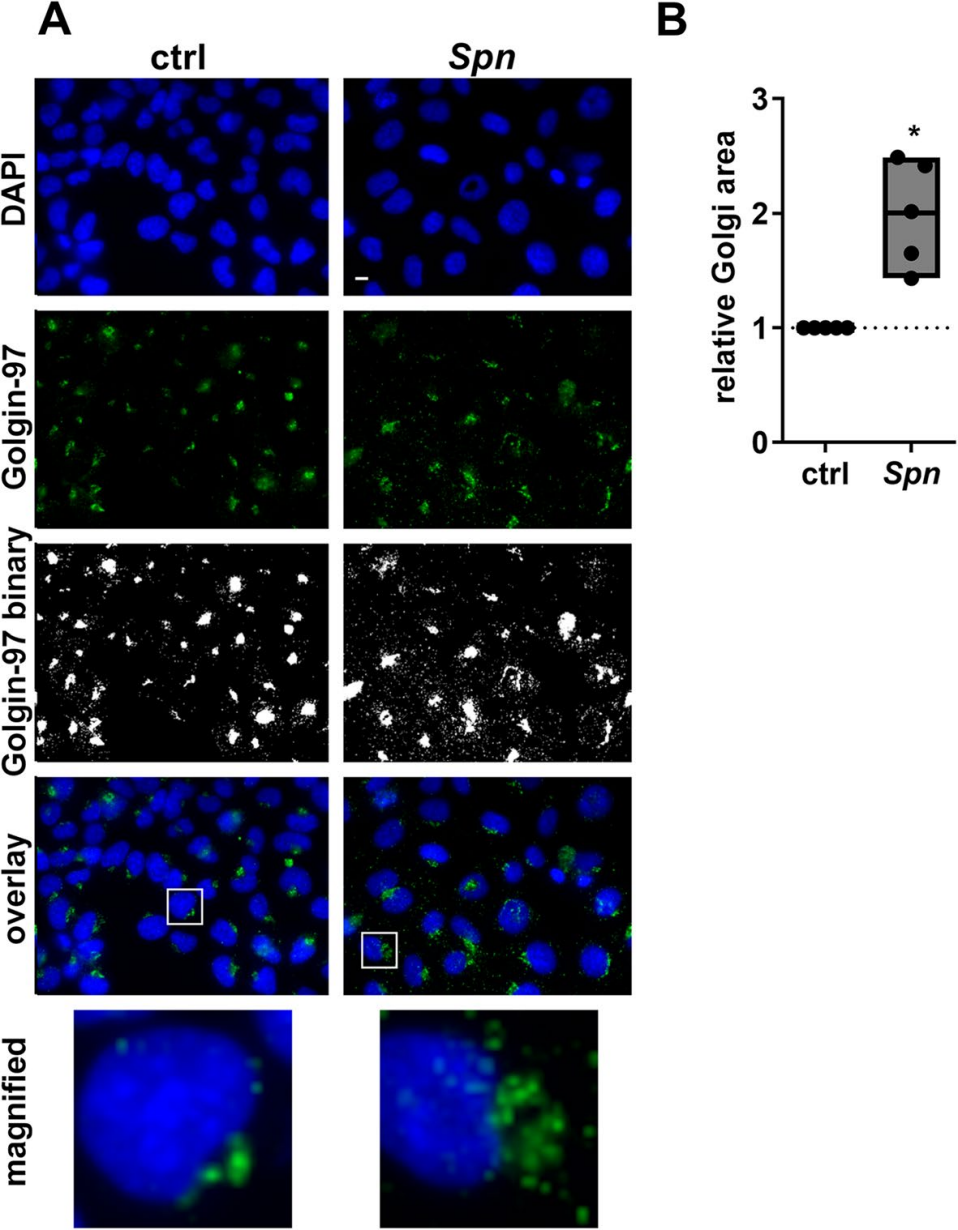
*Spn* infection disrupts the structural integrity of the Golgi apparatus. BEAS-2B cells were infected with *Spn* D39, MOI 1 for 16 h or left uninfected. Cells were fixed and fluorescence stained for Golgin-97 and the nucleus (DAPI). Binary images were generated and used to quantify the Golgi area. **A** Immunofluorescence images. Overlays were generated using the false-colored DAPI- and Golgin-97 images. **B** Quantification of the Golgi surface area, normalized to uninfected controls. (Statistics: paired two-tailed t-test; * = *p* < 0.05; Scale: 10 µm; at least 600 cells from 5 independent experiments were quantified)

We first assessed the effect of *Spn* D39 infection on Golgi integrity in bronchial epithelial cells using immunofluorescence staining for Golgin-97. Upon pneumococcal infection, we found a disruption of Golgi integrity as defined by a scattered fluorescence signal (Fig. 1A). Quantification of the total signal distribution showed an approximately two-fold increase of total Golgi area upon *Spn* infection, compared to uninfected controls (Fig. 1B). The same effect was observed when infecting BEAS-2B cells with the predominantly extracellular but highly aggressive pneumococcal strain TIGR4 (Figure S1).

### Deletion of the pneumococcal peroxidase rescues Golgi integrity

**Fig. 2:**
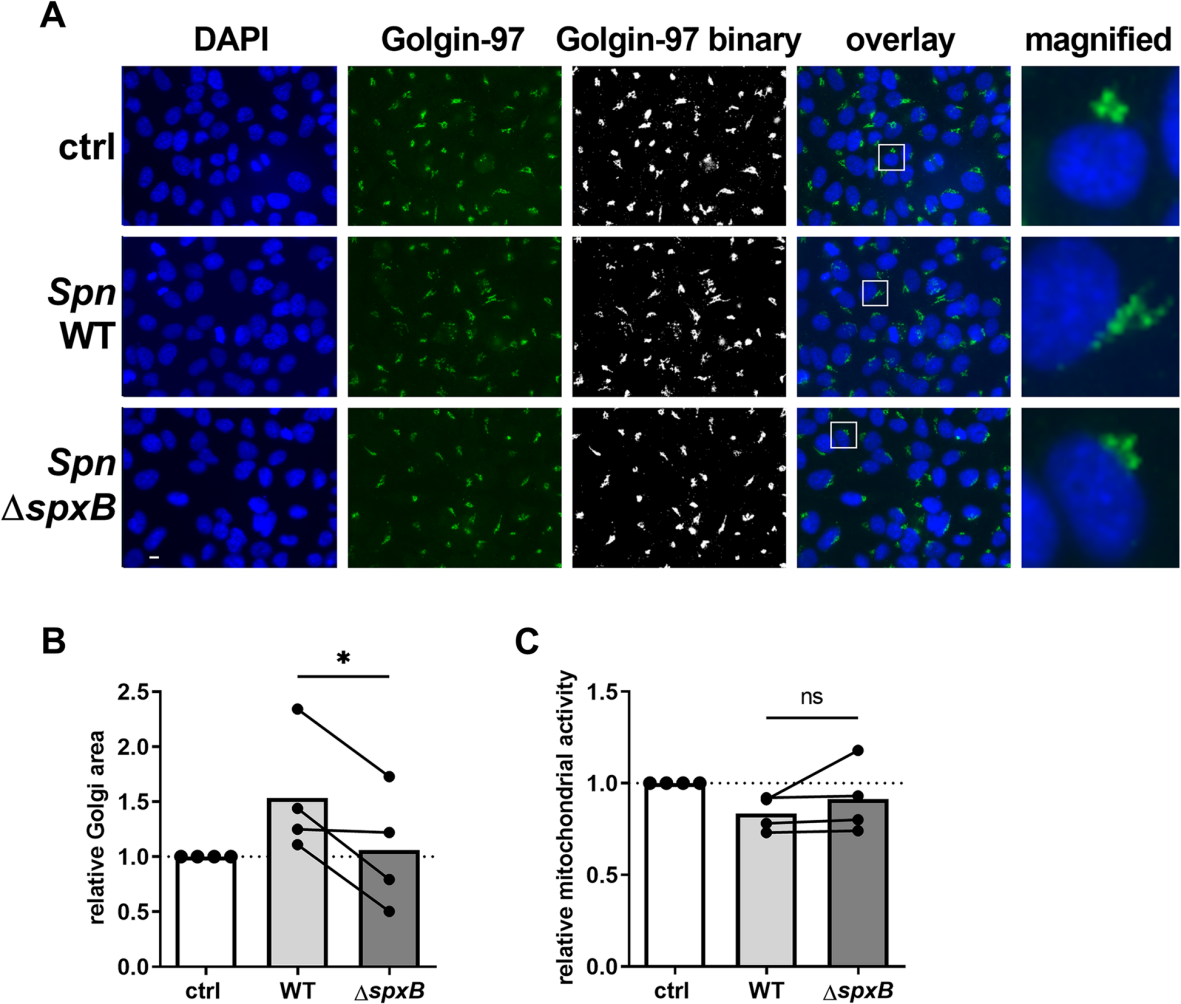
Deletion of the pneumococcal peroxidase rescues Golgi integrity. BEAS-2B cells were infected with *Spn* D39 WT or *Spn* D39 Δ*spxB* or left untreated. Cells were fixed and fluorescence stained for Golgin-97 and the nucleus (DAPI). **A** Immunofluorescence images of infected and uninfected cells. **B** Quantification of the Golgi surface area, relative to uninfected controls. **C** 16 h post infection, mitochondrial activity was assessed by MTT assay. (Statistics: paired two-tailed t-test for Δ*spxB* vs. WT; * = *p* < 0.05; Scale: 10 µm; at least 500 cells from 4 independent experiments were quantified)

As H_2_O_2_ is an important *Spn* virulence factor and was previously shown to induce Golgi scattering, we next aimed to determine if H_2_O_2_ is involved in *Spn*-mediated disruption of the Golgi apparatus. BEAS-2B cells were infected with a *Spn* D39 strain that is deficient of the H_2_O_2_-producing pneumococcal peroxidase (Δ*spxB*). Afterwards, cells were fluorescence stained and the Golgi surface area was quantified. The *ΔspxB* strain induced less Golgi scattering than wild type *Spn* D39 without affecting cell viability, supporting a H_2_O_2_-dependent mechanism of Golgi disruption (Fig. 2A, B, C). We further assessed, whether the pneumococcal pore-forming toxin pneumolysin is involved in Golgi fragmentation. However, deletion of pneumolysin did not significantly affect the ability of *Spn* to induce Golgi fragmentation, compared to the wild type strain (Figure S2).

### *H*_*2*_*O*_*2*_* inactivation prevents Golgi disruption*

**Fig. 3:**
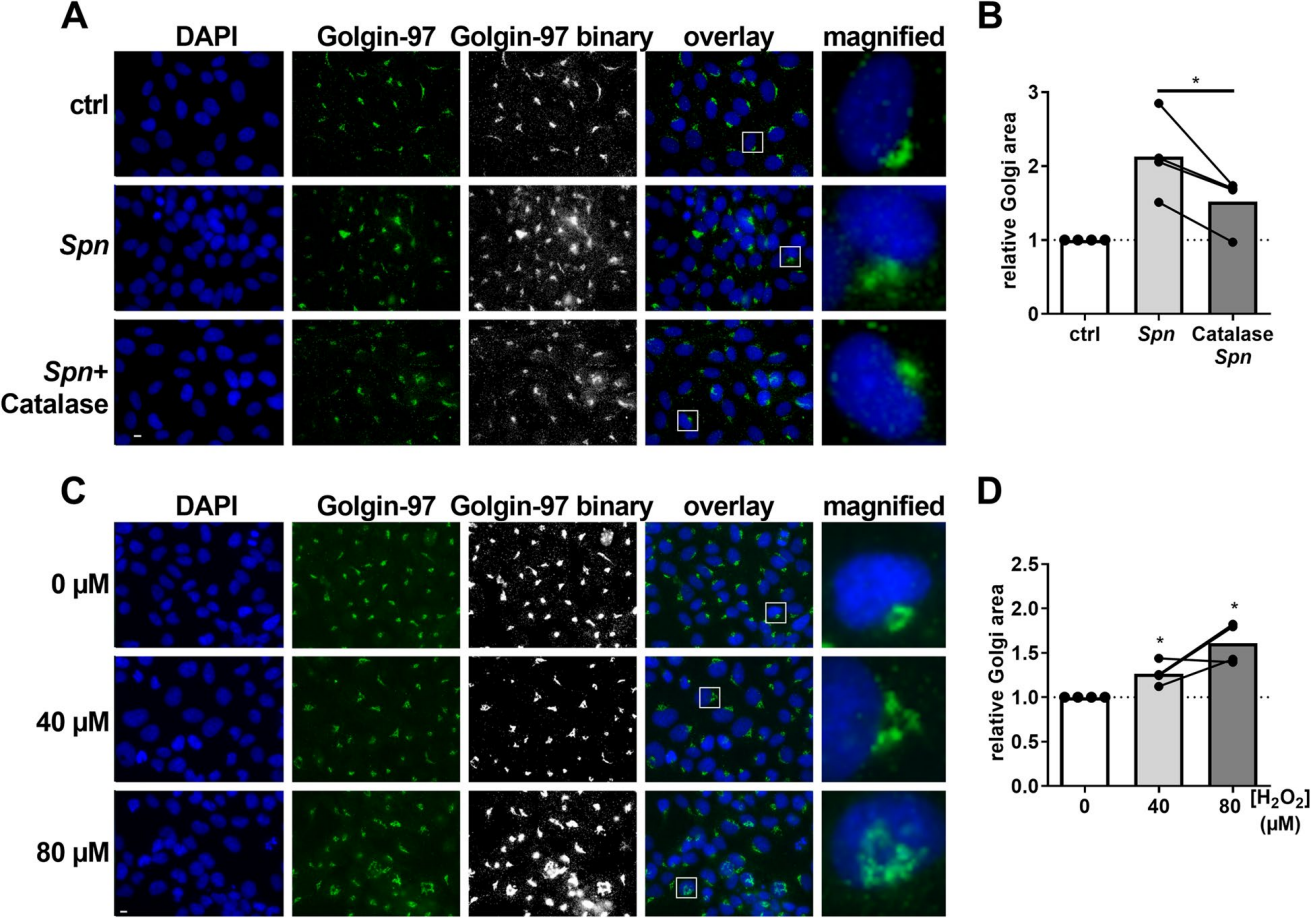
H_2_O_2_ production induces Golgi disruption. **A** BEAS-2B cells were infected with *Spn* D39 WT, MOI 1 for 16 h with or without addition of recombinant Catalase or left untreated. Cells were fixed and Golgin-97 and the nucleus stained. **B** Quantification of (**A**). **C** Cells were treated with H_2_O_2_ at indicated concentrations for 16 h. Cells were fixed and Golgin-97 and the nucleus stained. **D** Quantification of (**C**). (Statistics: paired two-tailed t-test; * = *p* < 0.05; Scale: 10 µm; at least 500 cells from 4 independent experiments were quantified)

To confirm the association of H_2_O_2_ production and Golgi scattering, we treated *Spn* D39 WT-infected BEAS-2B cells with the H_2_O_2_ degrading enzyme Catalase during infection (Fig. 3 A, B). Additionally, we treated cells directly with 40 or 80 µM H_2_O_2_ for 16 h prior to Golgi staining (Fig. 3 C, D). While Catalase treatment protected the Golgi structure upon *Spn* infection, H_2_O_2_ treatment induced Golgi disruption in a concentration-dependent manner. Taken together, these results support a H_2_O_2_ dependency of *Spn* induced Golgi scattering.

### Golgi disruption reduces bacterial adherence but increases replication

**Fig. 4:**
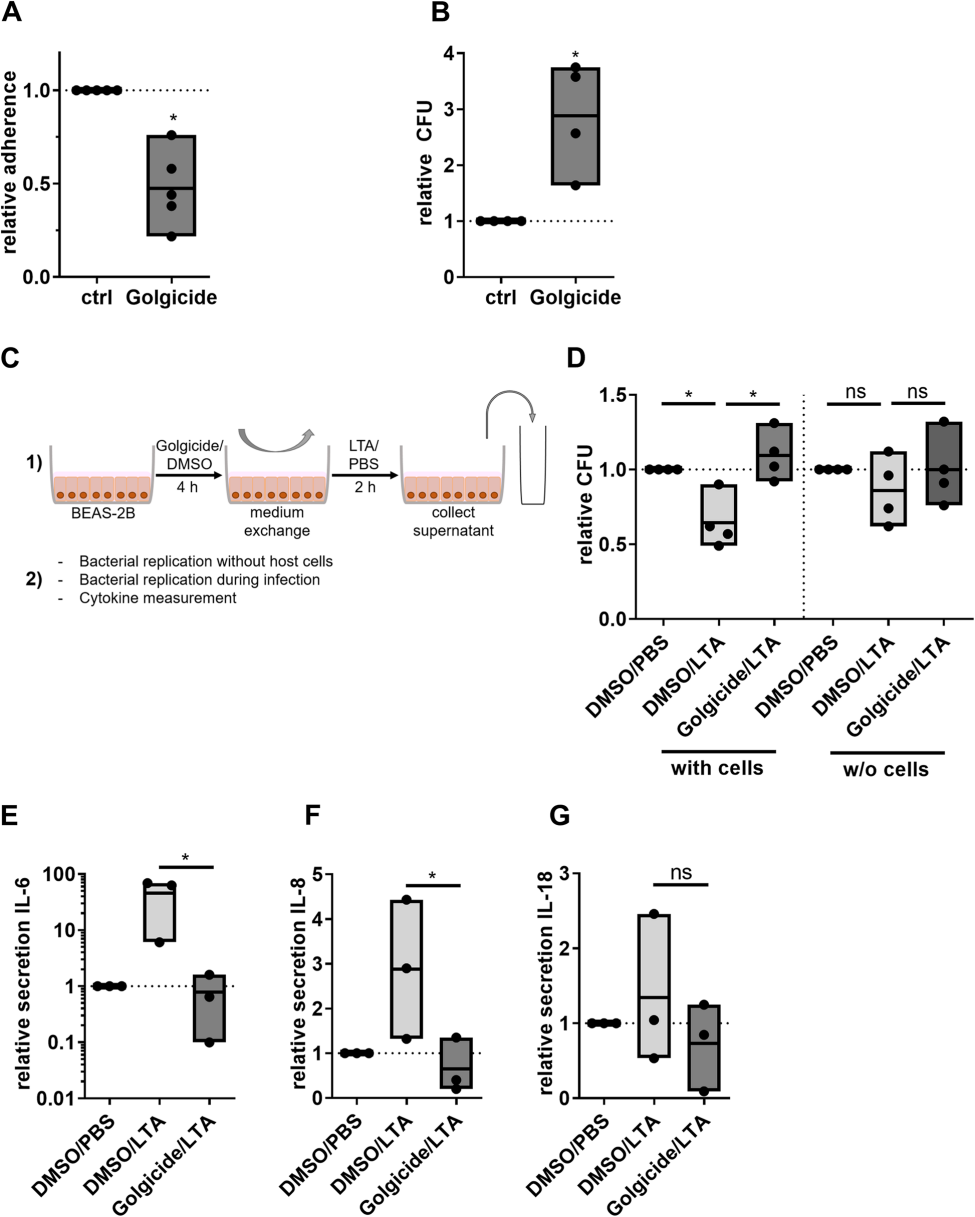
Golgi disruption reduces bacterial adherence but improves replication. **A** BEAS-2B cells were stimulated with Golgicide A, 4 µM or DMSO for 24 h. Afterwards, cells were infected with *Spn* D39, MOI 20 for 2 h. Cells were detached, lysed in distilled water and serial dilutions plated to determine numbers of adherent and internalized bacteria. **B** BEAS-2B cells were stimulated with Golgicide A or DMSO for 4 h. Afterwards, cells were infected with Spn D39, MOI 1 for 9 h and bacterial replication determined. **C**-**F** Cells were treated with Golgicide A or DMSO of 4 h. Medium was exchanged and cells were stimulated with LTA or PBS for 2 h and the supernatant collected. For infection experiments, cells were infected with *Spn*, MOI 1 for 16 h in infection medium consisting of 1:1 fresh cell culture medium and supernatant from 1). **C** Experimental setup. **D** BEAS-2B were infected with *Spn* D39, MOI 1 for 9 h in a 1:1 mixture of fresh cell culture medium and conditioned medium as indicated. Additionally, bacteria were cultivated in a host cell free mixture of fresh/conditioned medium for 9 h. Resulting CFU were determined. **E**, **F**, **G** Concentrations of IL-6, IL-8 and IL-18 were determined in Golgicide A/LTA-conditioned cell culture supernatants. (*N* = 3–4; Statistics: paired two-tailed t-test (**A**, **B**, **E**, **F**, **G**), One-way ANOVA with Fisher’s LSD (**D**); * = *p* < 0.05; ns = not significant)

As the Golgi plays an important role in mediating protein glycosylation, we hypothesized that pneumococcal interference with the Golgi might affect bacterial adherence to the host cell at the beginning of the infection process. To test our hypothesis, BEAS-2B cells were treated with Golgicide A to artificially induce Golgi fragmentation, prior to infection with *Spn*. Successful Golgi disruption and unaffected cell viability was confirmed by immunofluorescence microscopy and MTT assay (Figure S3). We found that Golgi disruption resulted in a 0.6-fold reduced bacterial adherence (Fig. 4A). Next, we assessed the influence of Golgicide A treatment on the bacterial replication during the infection process. Following Golgicide A treatment, bacterial replication in the cell culture media was increased by factor 2.8 compared to untreated controls, suggesting that Golgi disruption causes impaired secretion of antibacterial factors (Fig. 4B). To test whether these factors exert antibacterial effects directly or via a host cell-dependent mechanism, we induced pro-inflammatory activation of control or Golgicide A-treated cells using the TLR2-ligand LTA, followed by collection of the conditioned media (experimental setup illustrated in Fig. 4C). In absence of host cells, conditioned media did not influence bacterial replication, ruling out a direct antibacterial effect (Fig. 4D). However, when naïve cells were treated with conditioned media prior to infection, supernatant of LTA-stimulated, but not Golgicide A-treated cells reduced bacterial replication (Fig. 4D). In contrast, bacterial replication was rescued when supernatants were generated from cells treated with Golgicide A prior to LTA treatment. We therefore measured the quantity of anti-bacterial cytokines IL-6, IL-8 and IL-18. LTA treatment increased the secretion of IL-6 and IL-8 in cells with intact Golgi apparatus, whereas cytokine induction was abolished in Golgicide A treated cells (Fig. 4E, F, G). In summary, disruption of the Golgi apparatus leads to altered host cell glycosylation patterns and impaired cytokine secretion, which is associated with increased bacterial growth.

## Discussion

In this study, we observed that *Spn* infection interferes with Golgi structure and function in lung epithelial cells, thereby disturbing paracrine immune mechanisms. Fluorescence staining of the Golgi-associated protein Golgin-97 revealed a pneumococcal-induced structural disruption of the Golgi. Golgi fragmentation depended on the pneumococcal virulence factor H_2_O_2_. Artificial disruption of the Golgi apparatus by Golgicide A treatment influenced the infection process by inhibiting cytokine secretion, reducing bacterial adherence, but promoting replication.

The Golgi was previously reported as an important factor in the inflammatory response of epithelial cells [14]. Consequently, a range of intracellular pathogens were reported to interact with the Golgi. SARS-CoV2 was recently shown to disrupt the Golgi apparatus via a currently undefined mechanism [15]. Hepatitis C virus was shown to activate the Golgi-localized immunity related GTPase M, which phosphorylates the guanine nucleotide exchange factor GBF1, leading to disruption of the Golgi [16]. Upon bacterial infection, host-cell invading *Rickettsia rickettsii* and *Streptococcus pyogenes* were previously shown to disrupt Golgi structure [17, 18]. In contrast, encapsulated strains of *Spn* typically replicate extracellularly and only show minor tendencies to invade the host cell [19]. Thus, the present study is the first to show Golgi disruption in response to infection with extracellular bacteria. In neuroblastoma cells, H_2_O_2_, a common oxidant and virulence factor of *Spn,* was previously reported to induce Golgi fragmentation [12]. H_2_O_2_ treatment was shown to induce protease activity, leading to the degradation of the Golgi structure protein Ark1 and therefore Golgi fragmentation, as assessed by diffuse distribution of the tethering protein Golgin-97 [12, 20]. Here we hypothesized that Golgi disruption during *Spn* infection of epithelial cells is induced by a H_2_O_2_ dependent mechanism. We confirmed our hypothesis by demonstrating an induction of Golgi fragmentation in bronchial epithelial cells by H_2_O_2_ treatment, starting at 40 µM H_2_O_2_. This is likely to be relevant for the infection process, as H_2_O_2_ concentrations in liquid cultures of *Spn* were determined to reach up to 400 µM [5]. Treatment of infected cells with the H_2_O_2_-degrading enzyme catalase prevented Golgi fragmentation, and infection with the H_2_O_2_-deficient *Spn* strain *ΔspxB* failed to induce Golgi fragmentation, further supporting the involvement of H_2_O_2_*.* As a deletion of the pore-forming toxin pneumolysin did not significantly affect Golgi structure, we conclude that H_2_O_2_ is the main factor involved in pneumococci-induced Golgi disruption. Interestingly, catalase expression is a common feature of lung epithelial cells but reduced following Influenza A virus infection [21]. Thereby, catalase production might constitute a host cell defense mechanism against Golgi fragmentation and constitute a further mechanism by which initial Influenza A infection benefits a superinfection with *Spn.*

Beside its role in intracellular trafficking and organization of the cytoskeleton, the Golgi plays an important role in protein glycosylation. Disruption of the Golgi was linked to impaired protein glycosylation [22]. Bacterial adherence to the host epithelium is an important part of the pneumococcal infection process [23]. Mediated by various pneumococcal surface proteins and teichoic acids, *Spn* adheres to protein structures on the surface of the epithelial cells. Since extracellular protein domains are frequently glycosylated [24], we investigated whether artificial Golgi disruption by Golgicide A treatment [25] affects the adherence process. Indeed, upon Golgi disruption, the amount of adherent and internalized bacteria was reduced compared to untreated controls.

As the Golgi is well-established to be involved in the cellular antibacterial response [17], we next investigated whether Golgi disruption affects bacterial growth during the infection process. Indeed, bacterial replication increased if the epithelial cell Golgi apparatus was disrupted prior to infection. This effect was likely mediated by interference with Golgi-dependent secretory processes: after anti-bacterial activation with the Toll-like receptor 2-ligand LTA, epithelial cells secreted inflammatory cytokines IL-6 and IL-8 into their supernatant. These supernatants could in turn activate naïve epithelial cells, resulting in reduced bacterial replication upon *Spn* infection. When we experimentally disrupted the Golgi-apparatus of the secreting cells, this abolished both, cytokine secretion and supernatant-induced bacterial growth inhibition. This stands in accordance to previous results identifying a link between Golgi mediated membrane trafficking and cytokine secretion [26–28] and might constitute a mechanism for the previously reported inhibition of the inflammatory response in pneumococcal infections [29].

Epithelial cells form a first line of defense against invading pathogens. Therefore, these results might have important implications for the progression of a wide range of disease, e.g., infections with *S. oralis* or intestinal infections [29]. H_2_O_2_ production by commensal bacteria was furthermore reported to be an important part of the progression of inflammatory bowel disease [30]. The here reported H_2_O_2_-dependent Golgi fragmentation might constitute an additional mechanism of disease progression and H_2_O_2_ neutralization might form a readily available treatment option to slow disease progression and prevent tissue damage. However, in this study, we exclusively focused on the interference of *Spn*-produced H_2_O_2_ with the Golgi function in immortalized bronchial epithelial cells. Further work is needed to confirm this effect in more complex models. For instance, it would be of interest whether disruption of cytokine secretion following Golgi fragmentation affects the ability to recruit immune cells to the side of infection. The response of the innate immune system towards bacterial infection relies on secretory processes [31]. In particular, neutrophils were shown to rely on proper Golgi function to initiate antibacterial secretory processes [32]. Should *Spn*-mediated Golgi fragmentation occur in immune cells in addition to epithelial cells, this might constitute a new mechanism of immune evasion utilized by *Spn*. Lastly, by utilizing catalase to degrade H_2_O_2_, we identified an option to protect the Golgi apparatus during *Spn* infection in vitro. This provides a useful tool to study the effect of H_2_O_2_ on the Golgi and raises the question whether H_2_O_2_ neutralization might constitute a supportive treatment option during pneumococcal infection.

While animal experiments were beyond the scope of this study, further work should be done to confirm our results in vivo and assess the role of catalase as a potential Golgi-protective mechanism during lung infection. Therefore, mice should be infected with *Spn*, WT or Δ*spxB* to assess Golgi structure and secretion processes during infection. Infection of mice will furthermore serve as a useful models to investigate the effect of antioxidants during pneumococcal infection, with particular regard to Golgi structure and function.

## Conclusion

In summary, we report a novel interference of an extracellular bacterial pathogen with the Golgi apparatus, leading to an H_2_O_2_-dependent breakdown of Golgi structure and function. Our results further reveal a double-edged effect of Golgi fragmentation on the infection process. While initial infection might be hampered by a reduced bacterial ability to adhere to the host cell, in later stages Golgi disruption reduces the cellular defense against the pathogen. Thereby, our study provides evidence on a novel field of host–pathogen interaction during infections with the major human pathogen *Streptococcus pneumoniae.*

### Supplementary Information

**Additional file 1: Figure S1.** *Spn* TIGR4 infection disrupts the structural integrity of the Golgi apparatus. BEAS-2B cells were infected with *Spn* TIGR4, MOI 2 for 7 h or left uninfected. Cells were fixed and fluorescence stained for Golgin-97 and the nucleus (DAPI). Binary images were generated and used to quantify the Golgi area. A) Immunofluorescence images. Overlays were generated using the false-colored DAPI- and Golgin-97 images. B) Quantification of the Golgi surface area, normalized to uninfected controls. C) Mitochondrial activity after infection, assessed by MTT assay. (Scale: 10 µm; Statistics: paired two-tailed t-test; *N*=3-4; * = *p*<0.05; Scale: 10 µm; at least 300 cells from 3 independent experiments were quantified). **Figure S2.** Deletion of the pneumococcal pore-forming toxin pneumolysin does not affect Golgi integrity. BEAS-2B cells were infected with *Spn* D39 WT or *Spn* D39 Δply for 16 h or left untreated. Cells were fixed and fluorescence stained for Golgin-97 and the nucleus (DAPI). A) Immunofluorescence images of infected and uninfected cells. B) Quantification of the Golgi surface area, relative to uninfected controls. C) Mitochondrial activity, assessed by MTT assay. (Statistics: paired two-tailed t-test for Δ*ply* vs. WT; *N* = 4-5; * = *p*<0.05; Scale: 10 µm; at least 600 cells from 5 independent experiments were quantified). **Figure S3.** Golgicide A treatment efficiently disrupts the Golgi apparatus. A) BEAS-2B cells were stimulated with indicated amounts of Golgicide A for 4 h. Control cells were treated with DMSO. Afterwards, cells were fixated and stained for Golgin-97 and the nucleus. B) BEAS-2B cells were treated with Golgicide A, 4 µM for 4 h. Afterwards, medium was replaced with fresh medium containing 4 µM Golgicide and cells were incubated for additional 16 h. Control cells were treated with DMSO. After stimulation, mitochondrial activity was assessed by MTT assay. (Scale: 10 µM; *N*=3; Statistics: paired t-test (ns = not significant).

## Data Availability

All data generated and analysed during this study are included in this published article. Raw images are available from BS upon reasonable request.
