# Supplementary material for: Streptococcus pneumoniae disrupts the structure of the golgi apparatus and subsequent epithelial cytokine response in an H2O2-dependent manner
Source: Cell Commun Signal. 2023 Aug 17;21:208. doi: 10.1186/s12964-023-01233-x (PMC10436572; doi:10.1186/s12964-023-01233-x)
Supplement: Supplementary file 2 — Additional file 1: Figure S1. Spn TIGR4 infection disrupts the structural integrity of the Golgi apparatus. BEAS-2B cells were infected with Spn TIGR4, MOI 2 for 7 h or left uninfected. Cells were fixed and fluorescence stained for Golgin-97 and the nucleus (DAPI). Binary images were generated and used to quantify the Golgi area. A) Immunofluorescence images. Overlays were generated using the false-colored DAPI- and Golgin-97 images. B) Quantification of the Golgi surface area, normalized to uninfected controls. C) Mitochondrial activity after infection, assessed by MTT assay. (Scale: 10 µm; Statistics: paired two-tailed t-test; N=3-4; * = p<0.05; Scale: 10 µm; at least 300 cells from 3 independent experiments were quantified). Figure S2. Deletion of the pneumococcal pore-forming toxin pneumolysin does not affect Golgi integrity. BEAS-2B cells were infected with Spn D39 WT or Spn D39 Δply for 16 h or left untreated. Cells were fixed and fluorescence stained for Golgin-97 and the nucleus (DAPI). A) Immunofluorescence images of infected and uninfected cells. B) Quantification of the Golgi surface area, relative to uninfected controls. C) Mitochondrial activity, assessed by MTT assay. (Statistics: paired two-tailed t-test for Δply vs. WT; N = 4-5; * = p<0.05; Scale: 10 µm; at least 600 cells from 5 independent experiments were quantified). Figure S3. Golgicide A treatment efficiently disrupts the Golgi apparatus. A) BEAS-2B cells were stimulated with indicated amounts of Golgicide A for 4 h. Control cells were treated with DMSO. Afterwards, cells were fixated and stained for Golgin-97 and the nucleus. B) BEAS-2B cells were treated with Golgicide A, 4 µM for 4 h. Afterwards, medium was replaced with fresh medium containing 4 µM Golgicide and cells were incubated for additional 16 h. Control cells were treated with DMSO. After stimulation, mitochondrial activity was assessed by MTT assay. (Scale: 10 µM; N=3; Statistics: paired t-test (ns = not significant). [file 12964_2023_1233_MOESM1_ESM.docx]

# Supplemental Data


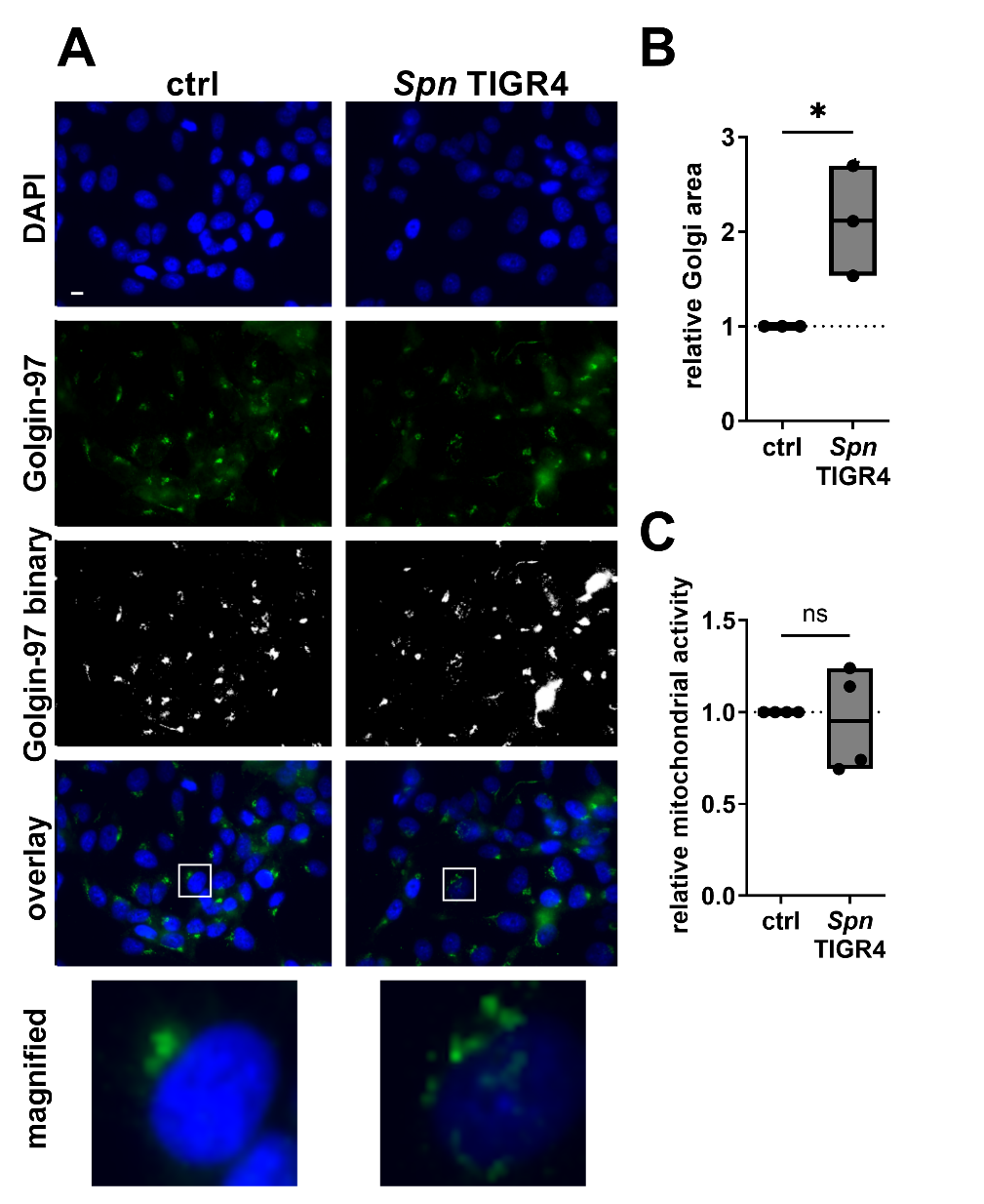


*Figure S1: Spn TIGR4 infection disrupts the structural integrity of the Golgi apparatus. BEAS-2B cells were infected with Spn TIGR4, MOI 2 for 7 h or left uninfected. Cells were fixed and fluorescence stained for Golgin-97 and the nucleus (DAPI). Binary images were generated and used to quantify the Golgi area. A) Immunofluorescence images. Overlays were generated using the false-colored DAPI- and Golgin-97 images. B) Quantification of the Golgi surface area, normalized to uninfected controls. C) Mitochondrial activity after infection, assessed by MTT assay. (Scale: 10 µm; Statistics: paired two-tailed t-test; N=3-4; * = p<0.05; Scale: 10 µm; at least 300 cells from 3 independent experiments were quantified)*


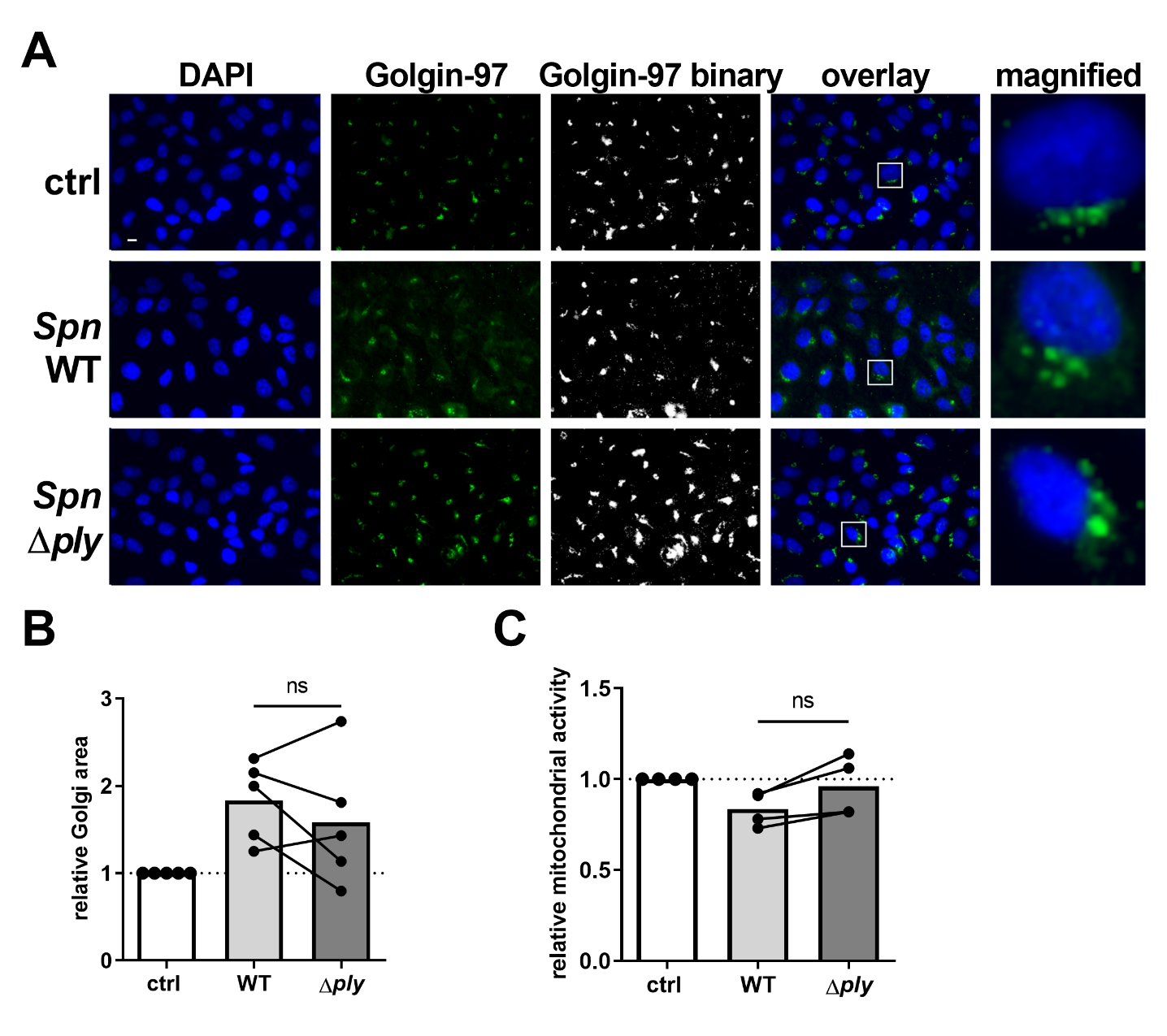


*Figure S2: Deletion of the pneumococcal pore-forming toxin pneumolysin does not affect Golgi integrity. BEAS-2B cells were infected with Spn D39 WT or Spn D39 Δply for 16 h or left untreated. Cells were fixed and fluorescence stained for Golgin-97 and the nucleus (DAPI). A) Immunofluorescence images of infected and uninfected cells. B) Quantification of the Golgi surface area, relative to uninfected controls. C) Mitochondrial activity, assessed by MTT assay. (Statistics: paired two-tailed t-test for Δply vs. WT; N = 4-5; * = p<0.05; Scale: 10 µm; at least 600 cells from 5 independent experiments were quantified)*


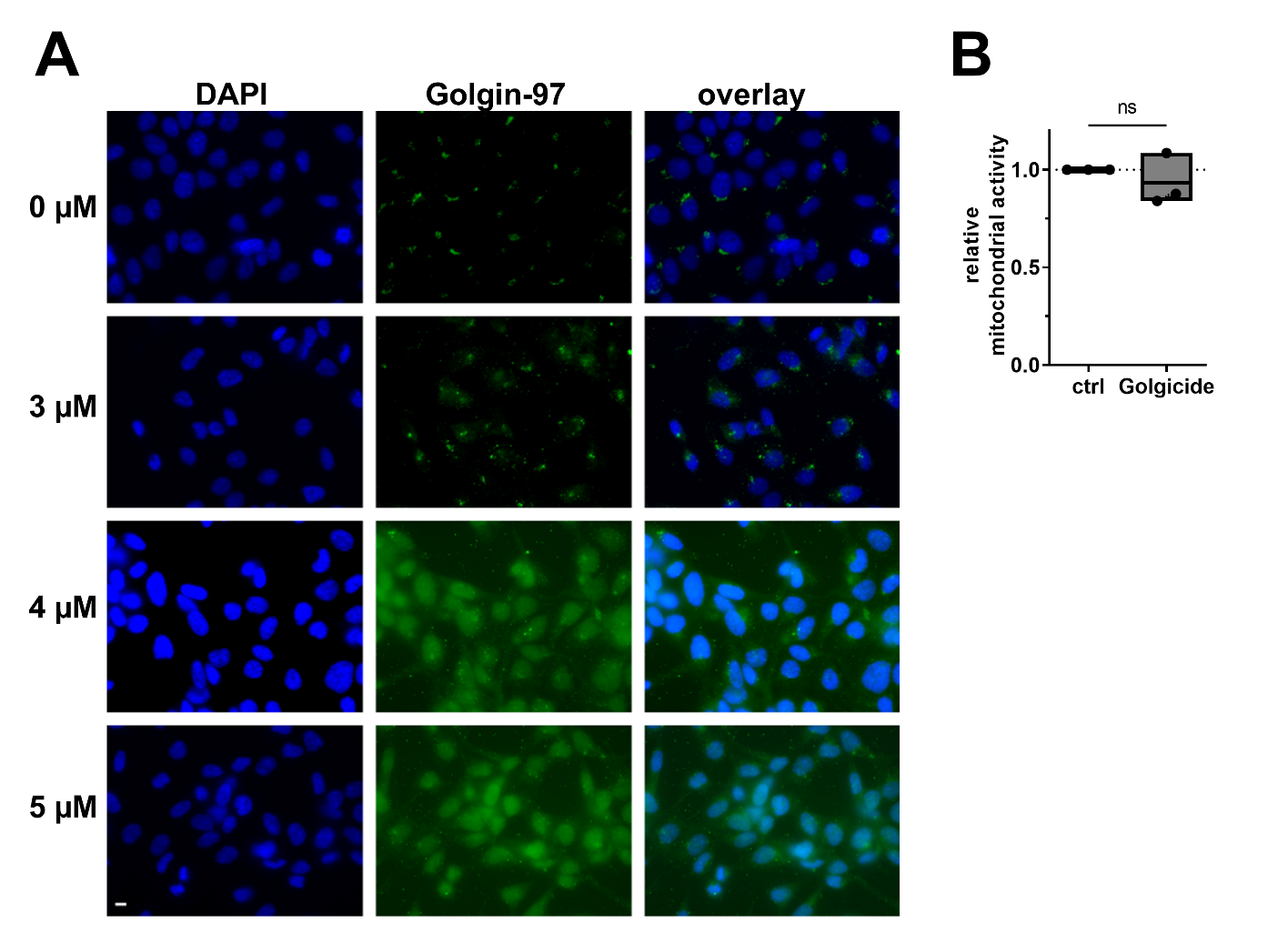


Figure S3: Golgicide A treatment efficiently disrupts the Golgi apparatus. A) BEAS-2B cells were stimulated with indicated amounts of Golgicide A for 4 h. Control cells were treated with DMSO. Afterwards, cells were fixated and stained for Golgin-97 and the nucleus. B) BEAS-2B cells were treated with Golgicide A, 4 µM for 4 h. Afterwards, medium was replaced with fresh medium containing 4 µM Golgicide and cells were incubated for additional 16 h. Control cells were treated with DMSO. After stimulation, mitochondrial activity was assessed by MTT assay. (Scale: 10 µM; N=3; Statistics: paired t-test (ns = not significant).
